# *Coreopsis tinctoria* Nutt ameliorates high glucose-induced renal fibrosis and inflammation via the TGF-β1/SMADS/AMPK/NF-κB pathways

**DOI:** 10.1186/s12906-018-2410-7

**Published:** 2019-01-10

**Authors:** Lan Yao, Jie Li, Linlin Li, Xinxia Li, Rui Zhang, Yujie Zhang, Xinmin Mao

**Affiliations:** 1 College of Traditional Chinese Medicine, Xinjiang Medical University, No. 4 Liyushan Park, Urumuqi, 830011 China; 2 College of Basic Medicine, Xinjiang Medical University, No. 393 Xinyi Street, Urumuqi, 830011 China; 3 Center of Analysis and Test, Xinjiang Medical University, No. 393 Xinyi Street, Urumuqi, 830011 China; 4 Institute of Traditional Chinese Medicine, Tianjin University of Traditional Chinese Medicine, No. 88 Yuquan Road, Nankai District, Tianjing, 300000 China

**Keywords:** Ethyl acetate extract of *Coreopsis tinctoria*, Anti-fibrosis and anti-inflammation, High glucose-treated mesangial cells, Mechanism

## Abstract

**Background:**

*Coreopsis tinctoria* Nutt is an ethnomedicine widely used in Xinjiang, China. It is consumed as a herbal tea by local Uyghur people to treat high blood pressure and diarrhea. Our previous study confirmed that the ethyl acetate extract of *Coreopsis tinctoria* (AC) had a protective effect on diabetic nephropathy (DN) in an in vivo experiment. Here we aim to elucidate the protective mechanism of AC and marein, the main ingredient in *Coreopsis tinctoria* on renal fibrosis and inflammation in vitro under high glucose (HG) conditions.

**Methods:**

A HG-induced barrier dysfunction model in rat mesangial cells (HBZY-1) was established. The cells were exposed to AC and marein and/or HG for 24 h. Then, the renal protective effects of AC and marein via transforming growth factor-β1 (TGF-β1)/Smads, AMP-activated kinase protein (AMPK), and nuclear factor kappa beta (NF-κB) signaling were assessed.

**Results:**

Both AC and marein suppressed rat mesangial cell hyperplasia and significantly attenuated the expression of HG-disrupted fibrotic and inflammatory proteins in HBZY-1 cells. It was also confirmed that AC and marein remarkably attenuated HG-induced renal inflammation and fibrosis by regulating the AMPK, TGF-β1/Smads, and NF-κB signaling pathways.

**Conclusion:**

These results indicated that AC and marein may delay the progression of DN, at least in part, by suppressing HG-induced renal inflammation and fibrosis. Marein may be one of the bioactive compounds in AC.

## Background

Diabetic nephropathy (DN), is one of the most prevalent and severe chronic microvascular complications of diabetes mellitus (DM), accounting for 30–47% of the cases of end-stage renal disease (ESRD) [1, 2] and is an important intervention target of ESRD [3]. As predicted by World Health Organization (WHO), due to the prevalence of diabetes and obesity, a rapid increase in DN is expected worldwide [4–6]. Although strict control of the degree of hyperglycemia, hyperlipidemia, and hypertension plays a vital role in the progression of DN, diabetic patients continue to develop nephropathy or other complications due to potential drug side effects and risk factors [7, 8]. Therefore, effective therapeutic approaches are needed.

The precise pathogenesis of DN is still not fully understood. Researchers agree that uncontrolled hyperglycemia in diabetic patients promotes renal inflammation, oxidative stress response and fibrosis, stimulates numerous pathologic molecular pathways, causes extracellular matrix accumulation, glomerular and tubular basement membrane thickening, extracellular and mesangial expansion [9], and contributes to renal fibrosis dysfunction [10]. Nuclear factor kappa beta (NF-κB), a central factor in inflammation can be triggered by hyperglycemia in vivo. Activated NF-κB translocates from the cytoplasm into the nucleus, and then promotes the expression of its target genes such as monocyte chemoattractant protein (MCP-1) and transforming growth factor-β1 (TGF-β1), which are important pro-inflammatory cytokines in DN progression [2, 11–13]. In addition, TGF-β1, a predominant pathogenic factor, regulates glomerular and tubulointerstitial fibrosis by the phosphorylation and activation of Smad2 and Smad3 as well as the canonical signaling pathway [14]. It is also known that AMP-activated kinase protein (AMPK) acts as a cellular energy homeostasis mediator and contributes to mesangial cell proliferation and fibrosis production [15].

Chinese herbal medicine (CHM), as an effective and safe therapeutic option, has received global attention. Evidence has confirmed that CHM can improve renal function by activating multiple signaling pathways [16, 17].

*Coreopsis tinctoria* Nutt is an ethnomedicine widely used in Xinjiang, China and in many other counties. It is consumed by local Uyghur people as a herbal tea to treat high blood pressure and diarrhea [18]. Previous studies have indicated that the dried flowers of the plant have anti-inflammatory, anti-antioxidant, anti-hyperlipidemic, and glycemic regulation activities [18–22].

In present study, the anti-inflammatory and anti-fibrotic effects of AC and its main component marein were further investigated in high glucose-treated rat glomerular mesangial cells. The multi-target mechanisms of AC and marein in vitro were also determined.

## Methods

### Chemicals and materials

High glucose Dulbecco’s Modified Eagle’s Medium (DMEM, Product code: 01–052-1), fetal bovine serum (FBS, Product code: 04–400-1), penicillin and streptomycin (Product code: 03–031-1) were purchased from HyClone (Logan, UT, USA). The cell counting kit (CCK8) was obtained from Boster (Wuhan, China; Product code: CK04). Marein was purchased from ChromDex (Irvine, CA, USA; Product code: ASB-00013126-005), antibodies against β-actin (Product code: ab8226), GAPDH (Product code: ab8245), Collagen IV (Product code: ab6586), fibronectin (FN, Product code: ab2413), TGF-β1 (Product code: ab92486), MCP-1 (Product code: ab7202), Smad4 (Product code: ab40759), NF-κB P-65 (Product code: ab16502), and ammonium pyrrolidine dithiocarbamate (PDTC, Product code: ab141406) were from Abcam (Cambridge, MA, USA). AMPK (Product code: 5832 T), p-AMPK (Product code: 2535 T), Smad2 (Product code: 8685 T), p-Smad2 (Product code: 8828 T), Smad3 (Product code: 9523 T), p-Smad3 (Product code: 9520 T), and 5-amino-4-imidazole carboxamide (AICAR) were from Cell Signaling (Danvers, MA, USA, Product code: 9944P). Dorsomorphin (Dor) was obtained from Sigma-Aldrich (St. Louis, MO, USA, Product code: P5499).

### Preparation of AC

The preparation of AC followed the procedure outlined in our previous study [23]. Briefly, flowers of *Coreopsis tinctoria* were harvested from Minfeng county, Hetian city, Xinjiang province of China. The species was identified by Professor Junping Hu, College of Pharmacy, Xinjiang Medical University. A voucher specimen of the plant material used in this study has been deposited in the herbarium of Ethnomedicine Research Institution in Urumuqi, Xinjiang province (No. 20120715278). The dried flowers of *Coreopsis tinctoria* (160 g) were ground to a powder and placed in 4 L of 55% ethanol for reflux extraction twice at 80 °C for 2 h. The extraction liquid was filtered and then concentrated using a rotary evaporator (R-210; Buchi, Essen, Germany) into 1 L of liquid extract. An equal volume of ethyl acetate was added to the liquid extract, which was then concentrated and spray-dried, yielding 7.04 g of AC powder. Then 0.1 g of AC powder was weighed and completely dissolved in 1 mL of dimethyl sulfoxide to prepare a 100 mg/mL stock solution of AC. After sterilization, the stock solution was diluted to different concentrations for use in the cell culture experiment [23].

### Cell culture and treatment

Rat glomerular mesangial cells (HBZY-1) were obtained from Boster (Wuhan, China, Product code: CX0130). The cells were incubated at 37 °C, under a 5% CO_2_ atmosphere and cultured in DMEM containing 10% FBS. Cells in passage 6–12 were used in the experiments. To determine the biological activity of AC and marein in vitro, HBZY-1 cells were starved in serum-free medium for 12 h. Normal control (NC) cells were cultured in DMEM medium containing 5.5 mM glucose. The model cells were treated/or not with different concentrations of AC and marein for 2 h, followed by exposure to 50 mM glucose in DMEM medium for 24 h [24].

### Cell viability assay

For the CCK8 assay, HBZY-1 cells were seeded in a 96-well plate at density of 1 × 10^5^/mL for 4–6 h to adhere. The cells were starved in serum-free medium for 12 h. NC cells were incubated in DMEM medium containing 5.5 mM glucose. Model cells were treated/or not with different concentrations of AC and marein for 2 h, followed by exposure to 50 mM glucose (high glucose; HG) in DMEM medium for 24 h and grouped as follows: the high glucose (HG), HG + AC 25 mg/mL, HG + AC 50 mg/mL, HG + AC 100 mg/mL, HG + AC 150 mg/mL, HG + marein 100 μM, HG + marein 200 μM, HG + marein 300 μM, or HG + marein 400 μM groups. Three replicates were included for each group. After treatment, the liquid supernatant was removed and 10 μL CCK8 reagent was added to each group for 2 h. The absorbance values were detected at 450 nm using a microplate reader (Thermo Fisher Multiskan FC, Waltham, MA, USA) following the manufacturer’s instructions.

### Western blotting

Cells were collected and extracted with RIPA (Thermo, Rockford, IL, USA) lysis buffer containing a protease and phosphatase inhibitor cocktail. The protein concentration of the cell lysate was determined using the bicinchoninic acid (BCA) protein assay kit (Thermo, Rockford, Pierce, USA) according to the manufacturer’s instructions. Protein lysate (30 μg) samples were electrophoresed on 10% sodium dodecyl sulfate polyacrylamide gel and transblotted onto a PVDF transfer membrane (Thermo, Rockford, Pierce, USA). The membranes were blocked in Tris-buffered saline with Tween (TBST) and 5% (*v*/v) nonfat milk for 1 h at room temperature, washed three times with TBST, and then incubated in rabbit anti-rat β-actin, GAPDH, collagen IV, FN, TGF-β1, MCP-1, Smad4, NF-κB P-65, AMPK, p-AMPK, Smad2, p-Smad2, Smad3, or p-Smad3 antibodies (1:1000) overnight at 4 °C. After washing three times with TBST, the membranes were combined with secondary anti-rabbit antibody (Invitrogen, Carlsbad, CA, USA) for 1 h at 37 °C. The protein bands were colored using the BCIP/NBT Substrate Kit (Thermo, Rockford, Pierce, USA) and the band densities were scanned and calculated with Quantity One v4.62 software [23].

### Immunofluorescence staining

HBZY-1 cells were cultured in four chamber slides at a density of 1 × 10^4^/mL for 4–6 h to adhere. After starvation for 12 h, the cells were divided into the NC group, HG group, HG + AC 150 mg/mL group, and HG + marein 400 μM group and treated for 24 h. Three replicates were included for each group. After treatment, the liquid supernatant was removed and the cells were then fixed with 4% paraformaldehyde for 30 min at room temperature followed by permeabilization with 4% PBS containing 4% Triton X-100 for 5 min and then rinsed three times with PBS. After blocking with 1% BSA or 30 min at room temperature, the cells were incubated with primary antibodies of FN, collagen IV, and MCP-1 overnight at 4 °C and subsequently incubated with secondary anti-rabbit antibody labeled with Alexa Fluor 488 (1:200; Abcam Cambridge, MA, USA). Cell nuclei were stained with DAPI (Sigma–Aldrich), and the cells were scanned with a fluorescent microscope (Leica SP8, Wetzlar, Germany) [24].

### Statistical analysis

All experiments were repeated at least three times and the data are presented as mean ± error of the mean. One-way analysis of variance was used to determine differences between multiple groups followed by Duncan’s multiple range test using SPSS 16.0 software (Chicago, IL, USA). Differences were considered statistically significant if the *p* value was less than 0.05.

## Results

### Effect of AC and marein on cell viability in high glucose (HG) treated HBZY-1 cells

**Fig. 1:**
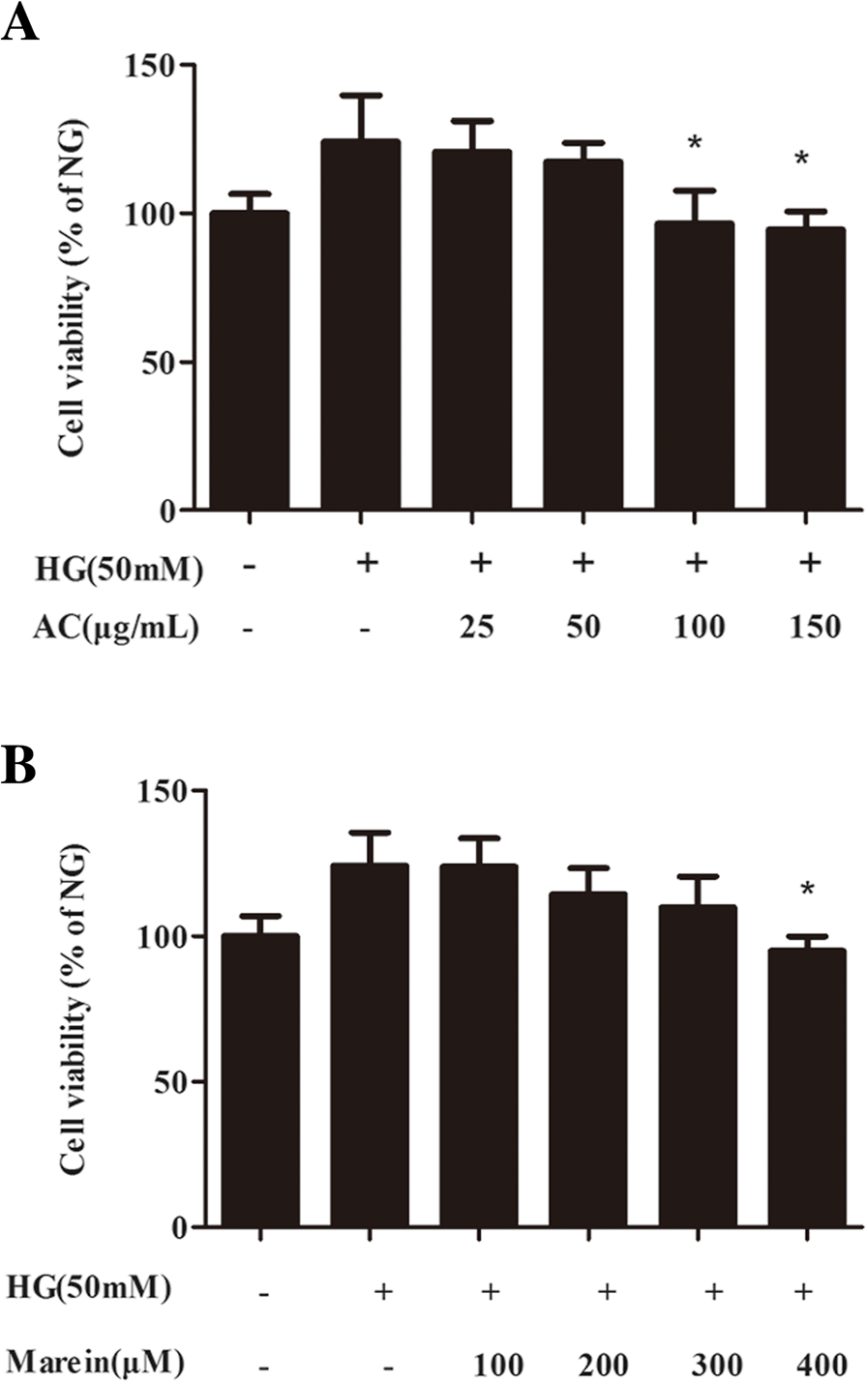
**a** Inhibitory effect of AC on cell proliferation in HG-treated HBZY-1 cells by the CCK8 assay. **b** Inhibitory effect of marein on cell proliferation in HG-treated HBZY-1 cells by the CCK8 assay. Note: Values of cell proliferation among the groups were analyzed as described previously in Materials and methods. Values were expressed as the mean ± SD, *n* = 4. ^#^*P <* 0.01 versus normal group; ^*^P < 0.05 versus control group; ^**^P < 0.01 versus control group

We assessed whether HG induced rat mesangial cell proliferation and whether AC and marein prevented cell proliferation. Compared with the NC group, cell proliferation increased by 24% in HG conditions (Fig. 1a and b). However, both AC and marein dose-dependently reversed this proliferation. In addition, AC at 100 and 150 mg/mL, and marein at 400 μM significantly inhibited cell proliferation and no cytotoxicity was observed, which indicated that AC and marein suppressed hyperplasia in rat mesangial cells.

### AC and marein decreased the expression of fibrotic proteins in HG-treated HBZY-1 cells

**Fig. 2:**
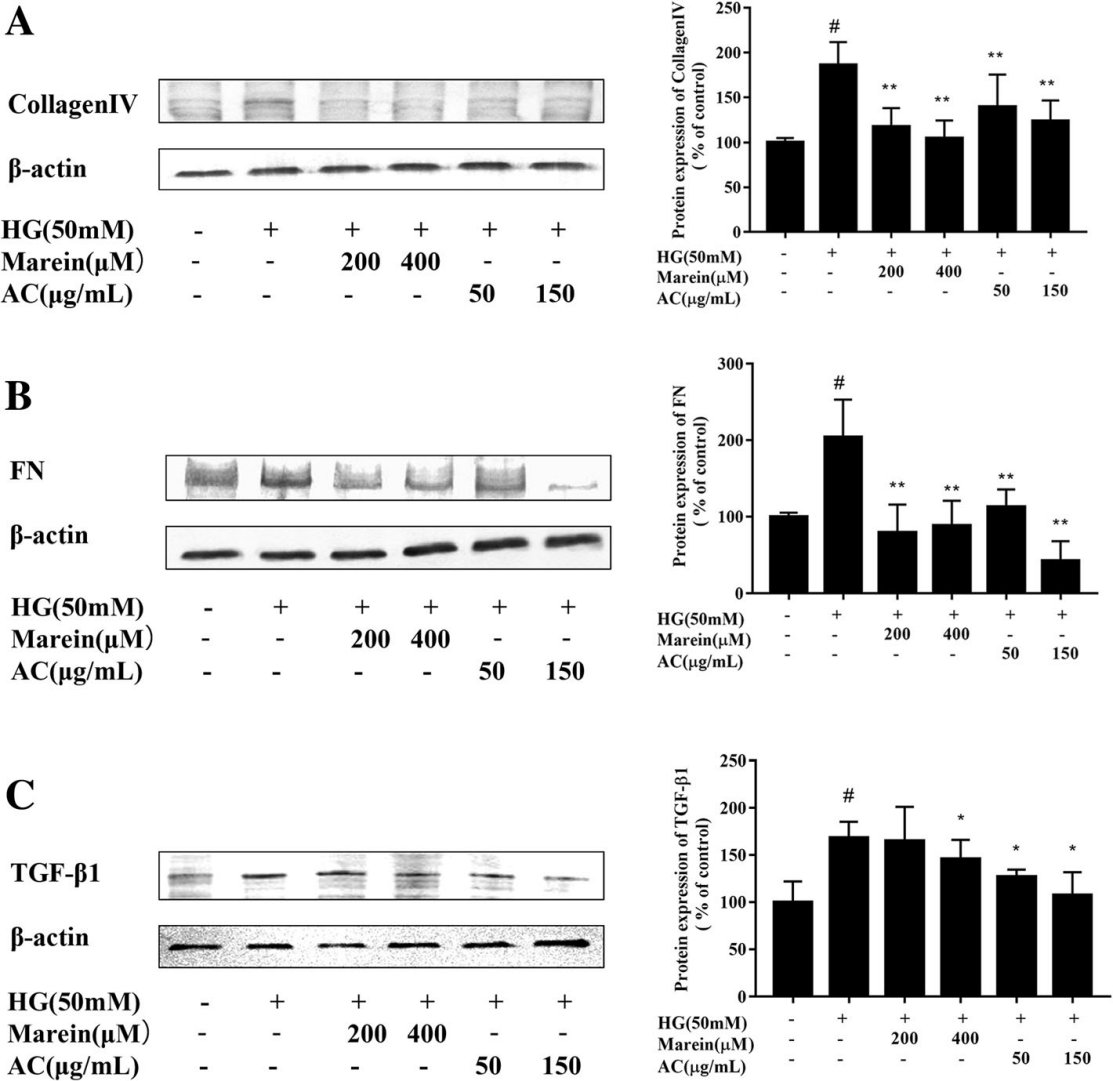
**a** Inhibitory effect of AC and marein on collagen IV expression in HG-treated HBZY-1 cells by western blots. **b** Inhibitory effect of AC and marein on FN expression in HG-treated HBZY-1 cells by western blots. **c** Inhibitory effect of AC and marein on TGF-β1 expression in HG-treated HBZY-1 cells by western blots. Note: Values were expressed as the mean ± SD, *n* = 4. ^#^*P <* 0.01 versus normal group; ^*^P < 0.05 versus control group; ^**^P < 0.01 versus control group

**Fig. 3:**
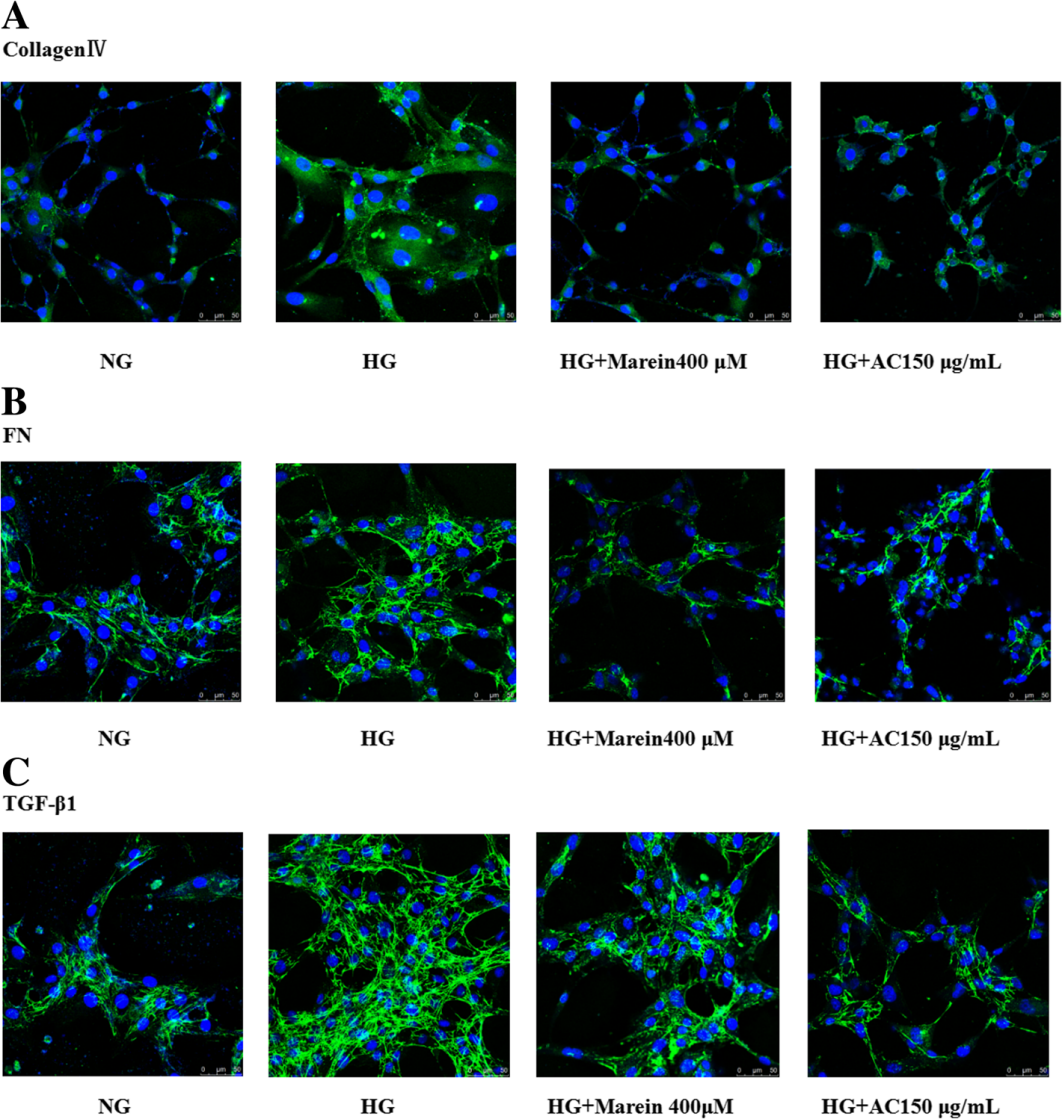
**a** Effect of AC and marein on collagen IV distribution in HG-treated HBZY-1 cells observed by immunofluorescence assay. **b** Effect of AC and marein on FN distribution in HG-treated HBZY-1 cells observed by immunofluorescence assay. **c** Effect of AC and marein on TGF-β1 distribution in HG-treated HBZY-1 cells observed by immunofluorescence assay. Note: Values were expressed as the mean ± SD, *n* = 4. ^#^*P <* 0.01 versus normal group; ^*^P < 0.05 versus control group; ^**^P < 0.01 versus control group

To assess the inhibitory effect of AC and marein on HG-induced mesangial fibrosis, the protein expression and distribution of fibrotic proteins such as collagen IV, FN, and TGF-β1 in mesangial cells were determined by western blots and immunofluorescence assays. HG significantly increased collagen IV, FN, and TGF-β1 protein expression and distribution in these cells (Figs. 2a-c and 3a-c). However, these increases were suppressed by both AC at 50 and 150 μg/mL and marein at 200 and 400 μM. These results suggested that AC and marein prevented HG-induced mesangial cell fibrogenesis.

### AC and marein regulated TGF-β/Smad signaling during mesangial cell fibrogenesis

**Fig. 4:**
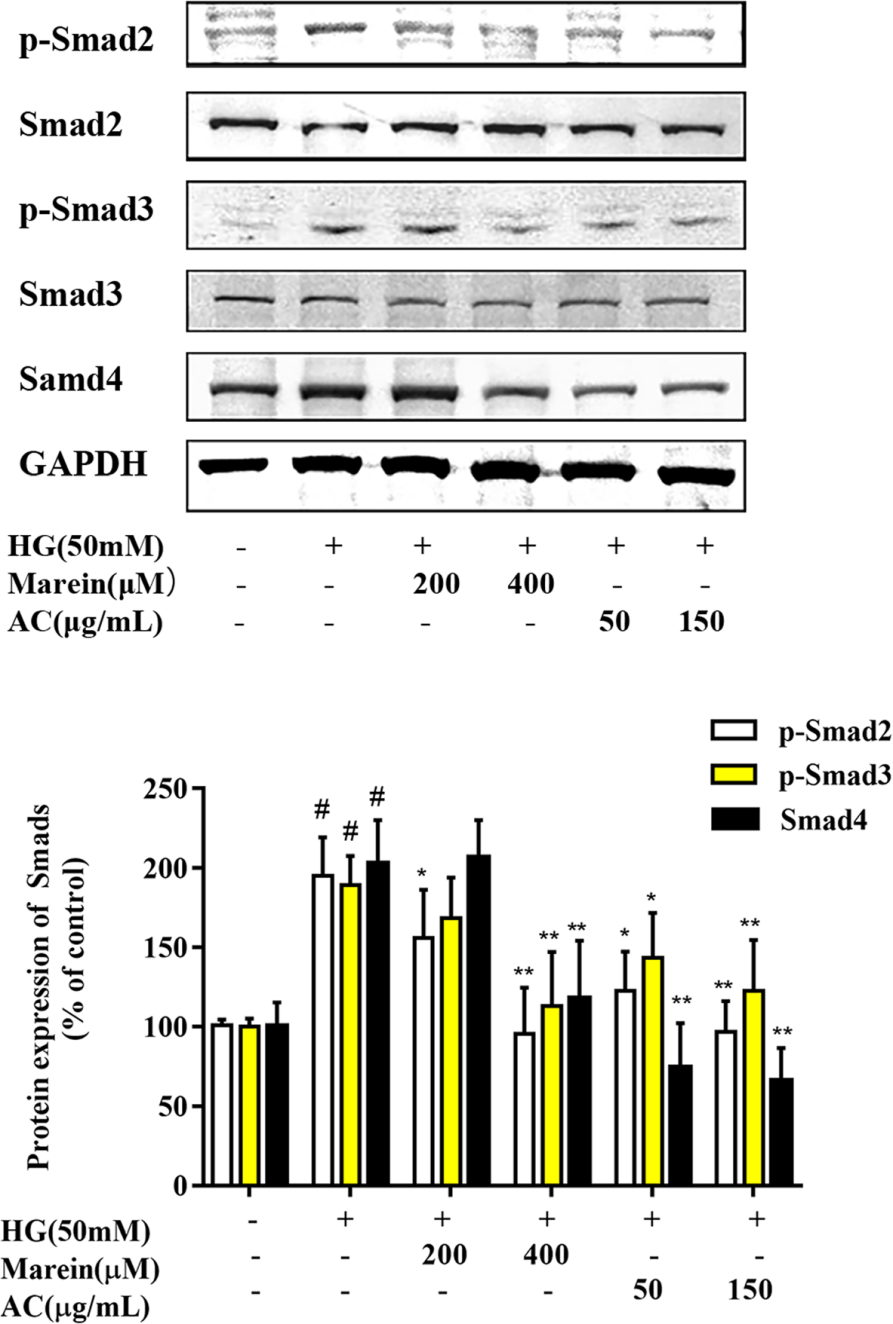
Effect of AC and marein on p-Smad2, p-Smad3 and Smad4 expressions in HG-treated HBZY-1 cells by western blots. Note: Values were expressed as the mean ± SD, *n* = 4. #*P <* 0.01 versus normal group; ^*^P < 0.05 versus control group; ^**^P < 0.01 versus control group

TGF-β/Smad signaling is highly activated in both experimental and human DN [25–27]. Smad2 and Smad3 are phosphorylated by TGF-β type I receptors, which bind to Smad4 to form oligomeric complexes, translocate into the nucleus and regulate renal fibrogenesis [28, 29]. It was confirmed that AC and marein can suppress TGF-β1 expression. To determine whether AC and marein inhibit renal fibrosis via TGF-β/Smad signaling, phosphorylated Smad2/3 and Smad4 were analyzed. Compared with the HG group, phosphorylated Smad2/3 and Smad4 were markedly decreased by AC and marein treatment (Fig. 4). These results were in partial agreement with those from our previous animal experiment [23].

### AC and marein inhibited mesangial cell fibrogenesis by regulating AMPK signaling in HG-treated HBZY-1 cells

**Fig. 5:**
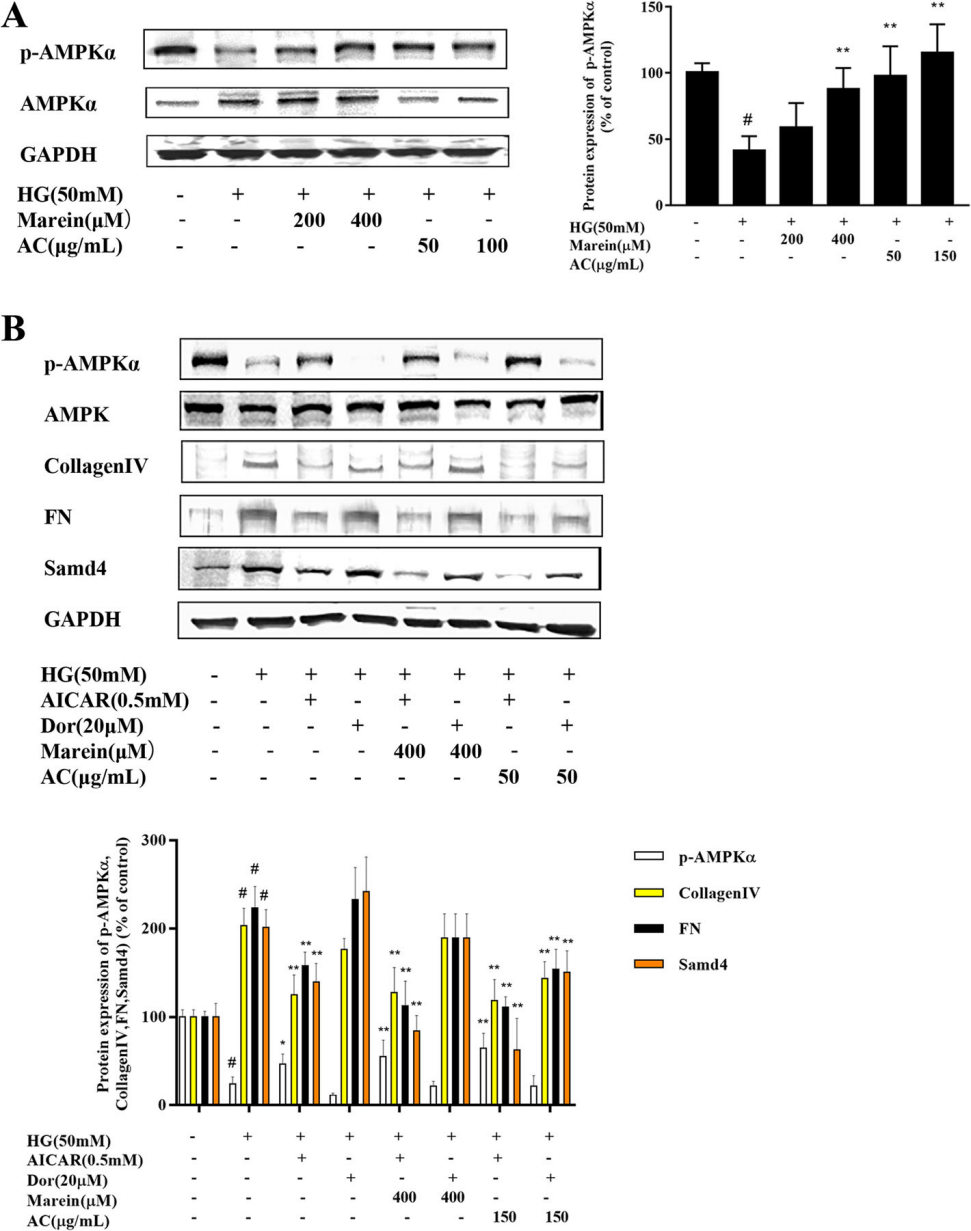
**a** Effect of AC and marein on p-AMPK expression in HBZY-1 cells by western blots. **b** HBZY-1 cells were pretreated with AC and marein for 2 h, and then stimulated with AICAR, an AMPK activator and Dorsomorphin (Dors) an AMPK inhibitor followed by NG or HG exposure for 24 h. Equal amounts of lysate were subjected to western blotting analysis to determine p-AMPK, collagen IV, FN, and Smad4 proteins. Note: Values were expressed as the mean ± SD, n = 4. ^#^*P <* 0.01 versus normal group; ^*^P < 0.05 versus control group; ^**^P < 0.01 versus control group

AMPK signaling plays an important role in diabetic renal fibrosis. A previous report indicated that AMPK inhibits TGF-β-induced matrix stimulation not by Smad2/3 phosphorylation but by inactivating Smad4 [30]. To determine whether AC and marein suppress the expression of fibrotic markers in HG-treated rat mesangial cells via AMPK signaling and Smad4 signaling, the activated form of AMPK, phosphorylated AMPK, was determined. Our results indicated that AC and marein significantly increased cellular phosphorylated AMPK expression (Fig. 5a). Further experiments showed that AICAR, an AMPK activator, increased the expression of phosphorylated AMPK, and decreased collagen IV, FN, and Smad4 expression. Dor, an AMPK inhibitor, reversed these changes. These results were partially consistent with those in a previous study [23]. The impact of AC and marein on HG-induced AMPK signaling was then investigated. It was observed that AC and marein reinforced the effect of AICAR on the upregulation of phosphorylated AMPK and downregulation of collagen IV, FN, and Smad4. In addition, AC and marein also reversed the activity of Dor. These results suggested that AC and marein decreased collagen IV and FN expression partially via AMPK/Smad4 signaling.

### AC and marein inhibited inflammation by regulating NF-κB signaling in HG-treated HBZY-1 cells

**Fig. 6:**
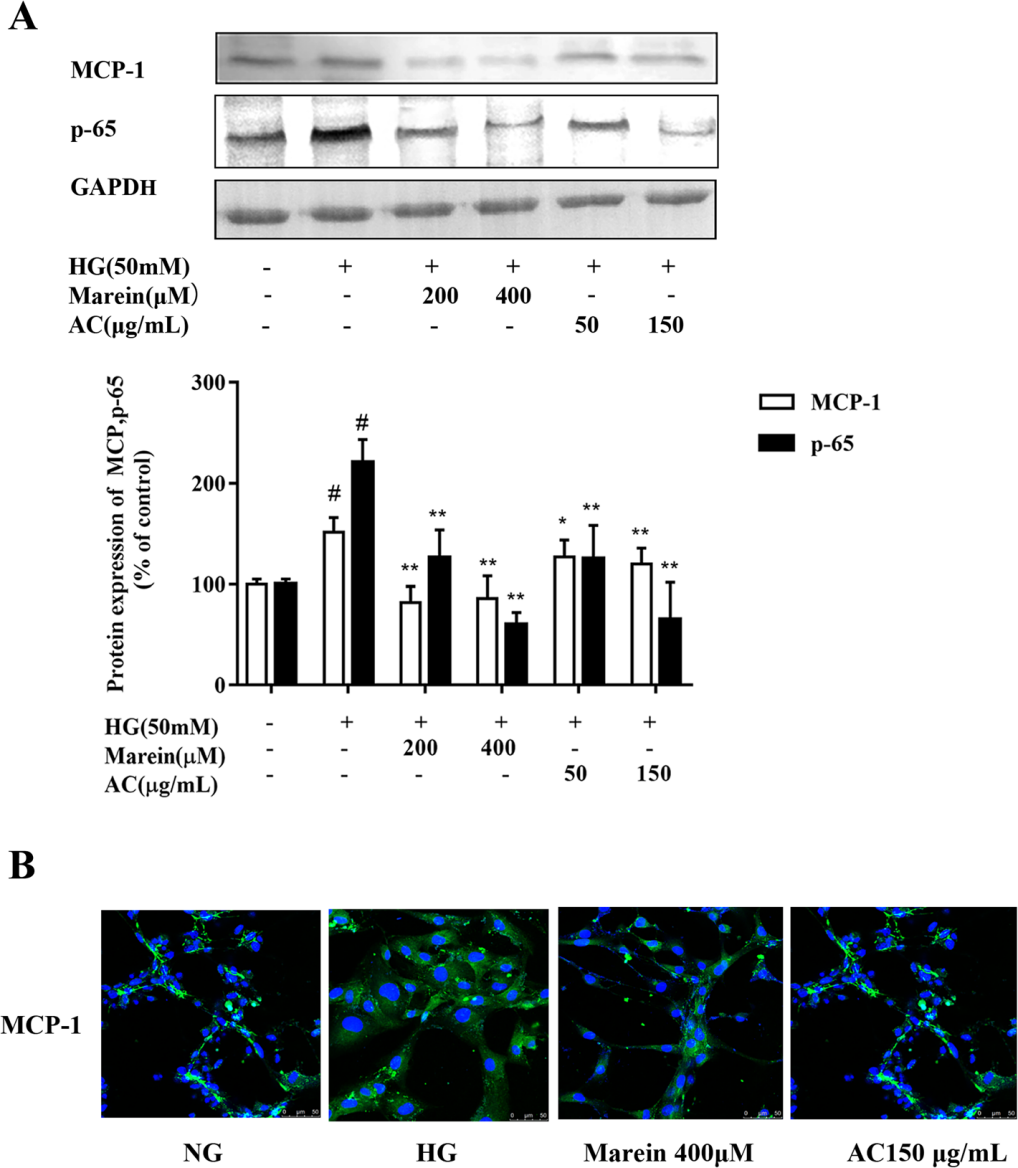
**a** Effect of AC and marein on MCP-1 expression in HG-treated HBZY-1 cells by western blots. **b** Effect of AC and marein on MCP-1 distribution change by immunofluorescence. Note: Values were expressed as the mean ± SD, n = 4. ^#^*P <* 0.01 versus normal group; ^*^P < 0.05 versus control group; ^**^P < 0.01 versus control group

**Fig. 7:**
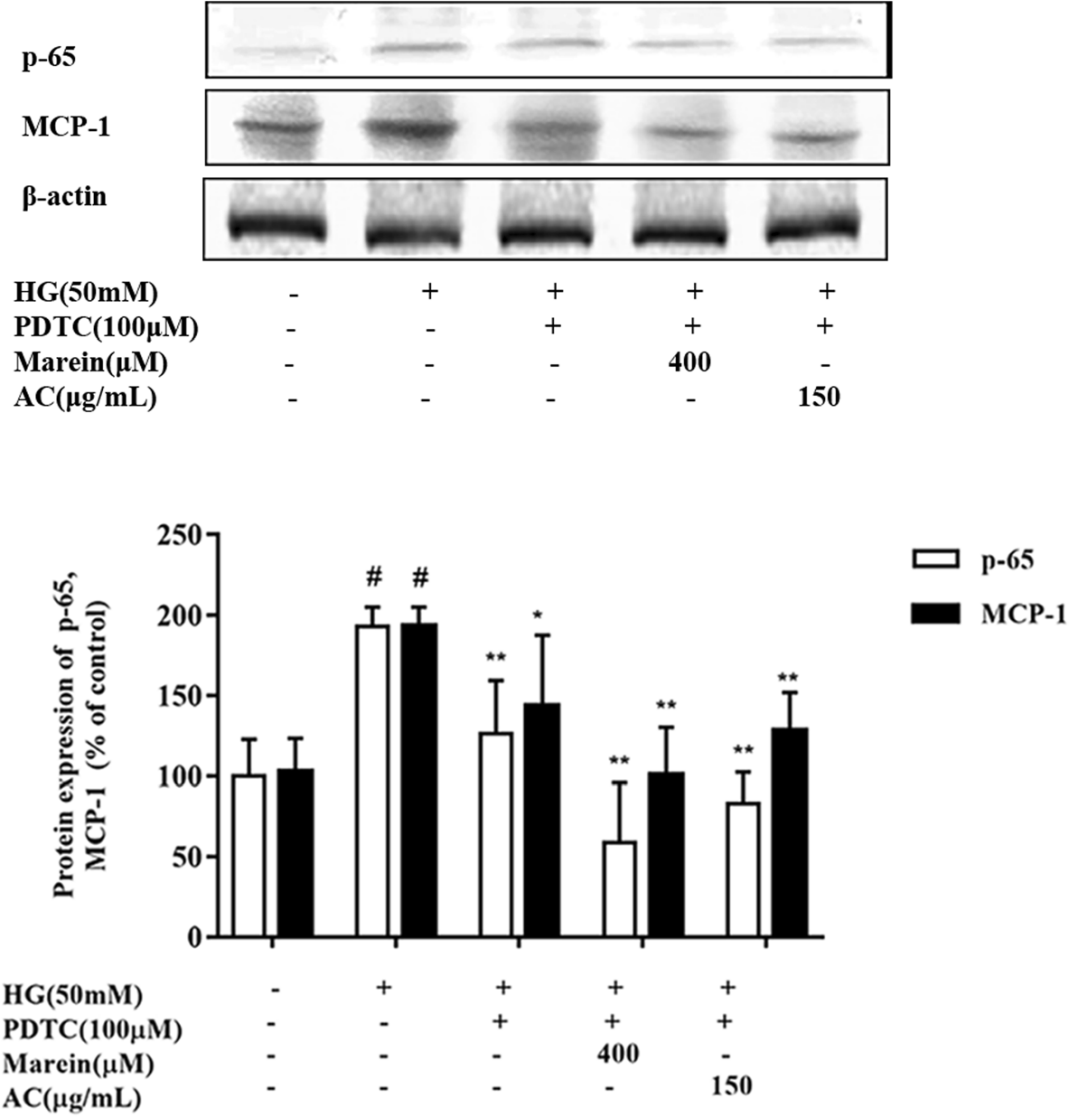
Effect of AC and marein on NF-κB signaling. HBZY-1 cells were pretreated with AC and marein for 2 h, and then stimulated with PDTC, an NF-κB inhibitor followed by NG or HG exposure for 24 h. Equal amounts of lysate were subjected to western blotting analysis to determine NF-κB P-65 and MCP-1. Note: Values were expressed as the mean ± SD, *n* = 4. #*P <* 0.01 versus normal group; ^*^P < 0.05 versus control group; ^**^P < 0.01 versus control group.

The immune system and chronic inflammation are both activated during the pathogenesis of DN [31]. MCP-1 is a vital cytokine in the renal inflammatory response, which is regulated by NF-κB signaling [32]. The present study revealed that AC and marein downregulated NF-κB, NF-κB P-65, and MCP-1 protein expression as well as the protein distribution of MCP-1 in HG-treated cells (Fig. 6a, b). The protein expressions of NF-κB P-65 and MCP-1 were further examined following treatment with PDTC, a NF-κB inhibitor, AC, and marein. The inhibitory effect of PDTC on NF-κB P-65 and MCP-1 protein expression was further enhanced by AC and marein to a certain degree (Fig. 7). Compared with PDTC treatment, marein at 400 μM decreased NF-κB P-65 and MCP-1 protein expression more, which indicated that AC and marein inhibited inflammation via NF-κB signaling.

### AC and marein inhibited mesangial cell fibrogenesis by regulating NF-κB signaling in HG-treated HBZY-1 cells

**Fig. 8:**
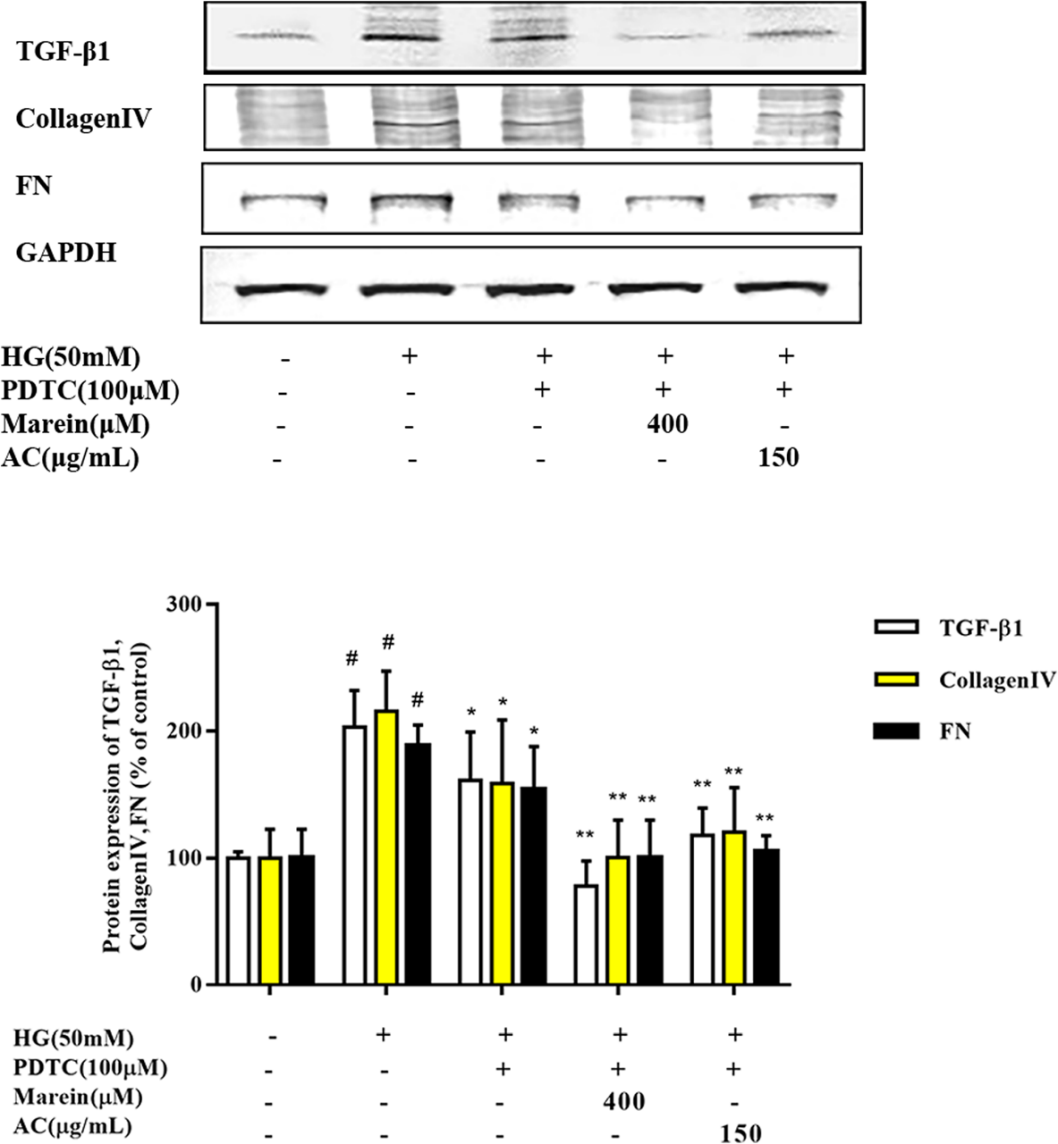
AC and marein ameliorated fibrotic markers by regulating NF-κB signaling. HBZY-1 cells were pretreated with AC and marein for 2 h, and then stimulated with PDTC, an NF-κB inhibitor followed by NG or HG exposure for 24 h. Equal amounts of lysate were subjected to western blotting analysis to determine TGF-β1, CollagenIV, and FN. Note: Values were expressed as the mean ± SD, *n* = 4. ^#^*P <* 0.01 versus normal group; ^*^P < 0.05 versus control group; ^**^P < 0.01 versus control group

To further define whether AC and marein inhibited mesangial cell fibrogenesis by regulating NF-κB signaling, we evaluated the inhibitory effect of AC and marein on the fibrosis markers TGF-β1, collagen IV, and FN by blocking NF-κB signaling. The results indicated that TGF-β1, collagen IV, and FN were reduced by PDTC treatment. Moreover, AC and marein strengthened the inhibitory effect on TGF-β1, collagen IV, and FN by PDTC. Compared with the PDTC treatment group, marein at 400 μM decreased TGF-β1 protein expression to a greater level (Fig. 8). These results confirmed that AC and marein inhibited TGF-β1, collagen IV, and FN protein expression via NF-κB signaling.

## Discussion

Natural bioactive compounds from plants have recently gained the attention of researchers due to their efficacy and low toxicity. For example, the chloroform extract of *Rumex hastatus* exhibited notable anti-tumor and anti-angiogenic activities. All the solvent fractions of *Rumex hastatus* were active against HeLa and NIH/3 T3 cell lines and most of the bioactive compounds were in the chloroform fraction [33, 34]. β-sitosterol isolated from *Polygonum hydropiper* has shown potential in the management of memory deficit disorders such as Alzheimer’s disease [35]. Despite the use of numerous therapeutic and preventive options for DN, the incidence of ESRD due to DN still remains high [36]. Conventional treatment is the main focus in controlling metabolic disorders and blood pressure; however, this treatment can cause various side effects, which may lead to secondary kidney injury and other uncertain symptoms. Nevertheless, CHM has advantages in preventing DN due to its synergistic effects with multiple compounds and reduced toxicity [17]. In our previous studies, we found that the ethyl acetate extract of *Coreopsis tinctoria* (AC), which is rich in flavonoids, prevented renal injury in streptozotocin (STZ)-induced diabetic rats. Moreover, we found that AC improved renal dysfunction and ameliorated renal inflammation and fibrosis possibly via the AMPK and TGF-β/Smad signaling pathways [23]. Based on these results, we observed that AC and the main flavonoid, marein, significantly inhibited the expression of fibrotic components FN, collagen IV, TGF-β1, and the pro-inflammatory cytokine MCP-1 in HG-treated rat mesangial cells. These results, which showed that AC and marein have therapeutic anti-fibrotic and anti-inflammatory effects in protecting DN in vitro, are consistent with animal experiments. We also attempted to define the molecular mechanism of AC and marein in renal fibrosis and inflammation in HG-treated rat mesangial cells. The data revealed that AC and marein decreased fibrosis possibly via TGF-β/Smad and AMPK signaling. In addition, renal inflammation was inhibited by AC and marein via NF-κB signaling. The relationship between inflammation and fibrosis was further determined by blocking NF-κB signaling. We found that the expression of fibrotic proteins was suppressed by blocking NF-κB signaling. These results were consistent with those of a previous study where renal inflammation was closely related to fibrosis [37]. Furthermore, AC and marein also inhibited renal fibrosis via NF-κB signaling.

TGF-β is known as a key pro-fibrotic regulator in driving renal fibrosis. The following three isoforms in the TGF-β family have been identified in mammals: TGF-β1, 2, and 3. Compared with other TGF-β isoforms, TGF-β1 is produced in all types of renal cells [38]. It has been demonstrated that TGF-β1 is a pro-fibrotic regulator in several ways. Firstly, TGF-β1 can independently induce fibrotic proteins such as FN and collagen I, Secondly, TGF-β1 extensively stimulates the phosphorylation of Smad2 and Smad3, and then activated Smad2 and Smad3 bond with Smad4 to form oligomeric complexes. The oligomeric complexes translocate into the nucleus, where they initiate the transcription of target genes including FN, collagen I, and Collagen IV [14, 39]. Therefore, TGF-β1 and Smads are considered therapeutic targets for renal fibrosis. In the present study, we showed that HG can increase TGF-β1 and activate downstream Smad2, Smad3, and Smad4 in rat mesangial cells. AC and marein reversed these effects. We speculated that AC and marein could inhibit renal fibrosis via a TGF-β/Smads-dependent or -independent pathway.

AMPK is an energy sensor that acts as a cellular energy homeostasis master switch by regulating multiple metabolic pathways [40]. AMPK is considered a crucial factor in tissues involved in development of the metabolic syndrome and diabetes [41]. In animal experiments, activation of AMPK ameliorated insulin resistance by improving glucose and lipid homeostasis. In addition, AMPK phosphorylates acetyl CoA-carboxylase and hydroxymethylglutaryl CoA reductase, which are the main downstream targets of AMPK. Phosphorylation of AMPK, which is involved in the rate-limiting steps of lipid homeostasis, can also promote fatty acid oxidation [42]. Recent studies have suggested that AMPK is ubiquitously and strongly expressed in the kidney and is correlated with diverse physiological and pathologic processes. It is well known that chronic exposure to glucose, lipids, and proteins during diabetes leads to toxic effects in various organs particularly the kidney [43]. This process inhibits AMPK activation which causes renal hypertrophy and fibrosis in hyperglycemic and hyperlipidemic conditions by regulating several pathways, and has been seen in both in vitro and in vivo experiments. In contrast, activation of AMPK suppressed both renal fibrosis and improved renal function in both in vitro and in vivo experiments [15, 44]. Therefore, AMPK could provide a potential approach to alleviate diabetic renal damage [45]. Metformin, an indirect activator of AMPK has been confirmed to improve renal function by downregulating the expression of renal fibrotic proteins [46, 47], which is in accordance with our previous animal experiment. In the present study, AC and marein promoted AMPK activation thereby preventing renal dysfunction. AICAR, an adenosine analogue which stimulates activation of AMPK is widely used in AMPK signaling research. AICAR targets genes associated with oxidative metabolism, angiogenesis, cell autophagy and glucose sparing, thereby improving diabetic kidney disease [48, 49]. Our results demonstrated that AICAR markedly suppressed the expression of renal fibrosis proteins and Smad4, and in turn Dor, a specific AMPK inhibitor, accelerated this expression. The finding that AMPK prevented renal fibrosis via Smad4 has been confirmed in a previous study [30]. Therefore, we speculated that the inhibitory effect of AC and marein on renal fibrosis is dependent on AMPK signaling via Smad4. In addition, we found that the inhibitory effect of AC and marein on Smad4 was via AMPK and TGF-β/Smads signaling.

In recent years, the role of inflammation in the progression of DN has been investigated. Activation of the immune system and chronic inflammation both occur in the pathogenesis of DN. Several studies have demonstrated that renal inflammation is regulated by the complex interaction of various factors. Cytokines, chemokines, adhesion molecules, nuclear factors as well as immune cells in both the glomerulus and tubules all play vital roles in the development of DN [50–52]. MCP-1 is an important chemokine in the renal inflammatory response. During the development of DN, the upregulation of MCP-1 increased the expression of adhesion molecules, and other pro-inflammatory cytokines by promoting monocyte and macrophage activation and infiltration into the glomerulus which exacerbated glomerular injury [53]. Various cell types can produce MCP-1 such as mesangial cells, tubular cells, podocytes and monocyte-macrophages [54]. MCP-1 expression can be detected in renal biopsies and MCP-1 excretion in urinalysis in DN patients [55]. Animal experiments have indicated that the deletion of MCP-1 reduced glomerular and interstitial injury [56, 57]. Moreover, MCP-1 inhibitors such as breviscapine and triptolide had a protective effect on DN by blocking the MCP-1 receptor in animal experiments [2]. Taken together, these findings demonstrate that MCP-1 could be a potential therapeutic target for DN treatment. Our results showed that both AC and marein significantly decreased HG-induced MCP-1 expression in HBZY-1 cells, which indicated that MCP-1 is an anti-inflammatory target of AC and marein in DN prevention.

NF-κB is a transcription factor and is activated by a wide variety of cellular responses to stimuli related to diabetes mellitus (DM) and its complications. Evidence has shown that NF-κB plays an important role in the development of DN [58]. Hyperglycemia, oxidative stress, and inflammation stimulate the activation of NF-κB via various signaling molecules. Increased activation of NF-κB is observed in most cell types in the kidney of diabetic patients and enters the nucleus to promote pro-inflammatory genes and cytokines such as MCP-1 and IL-6 (interleukin-6) and leads to renal apoptosis. NF-κB also accelerates renal fibrosis by activating cellular matrix accumulation of FN and collagen IV [26, 59]. In our experiments, AC and marein inhibited NF-κB activation via NF-κB and NF-κB P-65 expression. Interestingly, we found that suppression of NF-κB decreased MCP-1, FN and collagen IV expression, and both AC and marein strengthened these suppressive effects. These results indicated that the anti-inflammatory and anti-fibrotic effects of AC and marein were via NF-κB signaling.

Further experiments on the anti-inflammatory and anti-fibrotic mechanisms of AC and marein showed that the mechanisms involved the TGF-β1/Smads, AMPK, and NF-κB signaling pathways. Although marein is the main constituent in AC, the activity of many other constituents in AC still need to be determined in order to identify which are the most effective fractions or compounds. As *Coreopsis tinctoria* is rich in flavonoids, further studies on the the molecular targets of purified flavonoids from AC and their main constituents require determination.

## Conclusions

In summary, we conclude that AC from *Coreopsis tinctoria* has protective effects on DN, at least in part, by suppressing HG-induced renal inflammation and fibrosis, and marein is an active compound in AC. The activities of purified flavonoids in the AC and other active components merit further study.

## Abbreviations

AC: Ethyl acetate extract of *Coreopsis tinctoria* Nutt
AMPK: AMP-activated kinase protein
BCA: Bicinchoninic acid
CCK8: Cell counting kit
CHM: Chinese herbal medicine
DM: Diabetes mellitus
DMEM: Dulbecco’s Modified Eagle’s Medium
DN: Diabetic nephropathy
Dor: Dorsomorphin
ESRD: End-stage renal disease
FBS: Fetal bovine serum
FN: fibronectin
HBZY-1: Rat mesangial cells
HG: High glucose
MCP-1: Monocyte chemoattractant protein
NC: Normal glucose control
NF-κB: Nuclear factor kappa beta
STZ: Streptozotocin
TGF-β: Transforming growth factor-β
WHO: World Health Organization
